# Is robotic pancreaticoduodenectomy non-inferior to open pancreaticoduodenectomy in patients with high PD-ROBOSCORE?

**DOI:** 10.1007/s00464-025-11550-6

**Published:** 2025-02-18

**Authors:** Carolina Gonzalez-Abos, Filippo Landi, Claudia Lorenzo, Samuel Rey, Francisco Salgado, Fabio Ausania

**Affiliations:** 1 Department of HBP and Transplant Surgery, Hospital Clínic de Barcelona, C. Villarroel, 170, 08036 Barcelona, Spain; 2 University of Barcelona, Barcelona, Spain; 3 Gene Therapy and Cancer, IDIBAPS, Barcelona, Spain

**Keywords:** Robotic surgery, Pancreaticoduodenectomy, Complications, High difficulty

## Abstract

**Introduction:**

Robotic pancreaticoduodenectomy (RPD) is associated with technical challenges that may result in intraoperative and postoperative complications. Some previous reports and the recently published PD-ROBOSCORE describe several factors associated with an increased difficulty. The aim of this study is to investigate whether difficult RPD patients have a better outcome when operated by open approach (OPD).

**Methods:**

All patients undergoing robotic and open PD from January 2020 to June 2024 with high PD-ROBOSCORE were included. Preoperative pancreatitis and/or cholangitis, and tumor contact with PV-SMV were also analysed. Outcomes of RPD vs OPD were compared.

**Results:**

45 RPD and 57 OPD patients with high PD-ROBOSCORE were considered for this study. Median age was 68.5 years (68 RPD vs 65 OPD; p = 0.25), median BMI was 27 kg/m^2^ (27 RPD vs 28 OPD; p = 0.13), 65.6% of patients were male (60.0% RPD vs 70.2% OPD; p = 0.15) and median PD-ROBOSCORE was 10 (10 RPD vs 9 OPD, p = 0.145). POPF occurred in 37.2% (40.0% RPD vs 35.1% OPD; p = 0.668), CD ≥ 3 was 25.4% (28.8% RPD vs 22.8% OPD; p = 0.477), median CCI was 20.9 (20.5 RPD vs 20.9 OPD; p = 0.752), reoperation rate was 17.6% (15.5% RPD vs 19.3% OPD; p = 0.496). Hospital stay was 15 days (16 RPD vs 13 OPD; p = 0.583). Of patients developing POPF; 76.3% had soft pancreas, 84.2% had pancreatic duct ≤ 2 mm and 97.2% had BMI ≥ 25.

**Conclusion:**

RPD seems to be non-inferior to OPD in patients with increased technical complexity. Most of these complications are related to fistula risk factors (high BMI, soft pancreas and small pancreatic duct) and not directly related with other technical difficulty factors.

Pancreaticoduodenectomy (commonly known as the Whipple procedure) is one of the most complex surgical procedures, but not all Whipple procedures are the same [1, 2]. These surgeries exhibit varying levels of complexity depending on factors such as patient anatomy, comorbidities, and tumor characteristics. Recognizing this variability is crucial, as it highlights the need to compare surgical techniques and approaches only between groups of similar complexity [3].

The complexity of pancreatic surgery poses significant risks, often resulting in increased morbidity, mortality, and poorer outcomes, even in high-volume centers where expertise is elevated. Accurate stratification of difficulty levels can help guide surgical planning and decision-making [4], optimizing outcomes and minimizing complications. Traditionally, parameters such as intraoperative blood loss and operative time have been used to estimate surgical difficulty, but they remain subjective and lack standardization [5].

To address this gap, the newly described PD-ROBOSCORE provides an opportunity to stratify pancreaticoduodenectomies based on anticipated difficulty levels [4]. By incorporating patient-specific and surgical variables, this scoring system allows for objective comparisons between surgical methods, particularly between robotic and open approaches. These comparisons are essential for tailoring surgical strategies, advancing preoperative planning, and improving patient outcomes.

This study evaluates the outcomes of high-difficulty robotic pancreaticoduodenectomies compared to traditional open approaches. By leveraging the PD-ROBOSCORE, it aims to offer insights into the relative safety and efficacy of robotic techniques in managing these complex cases [4].

## Methods

All patients undergoing robotic and open PD from January 2020 to June 2024 were considered for this study. The robotic pancreaticoduodenectomy (RPD) program at our institution was initiated in January 2022, and currently, 80% of pancreaticoduodenectomies are performed using the robotic approach. The decision to proceed with robotic surgery is influenced by factors such as the need for vascular resection, the severity of obesity, the presence of prior major supramesocolic surgeries, and the availability of the robotic system. To ensure a sufficient number of patients operated on via the open approach with high PD-ROBOSCORE, we included patients treated between 2020 and 2022 in this study. This approach allowed us to establish a robust control group of open pancreaticoduodenectomy (OPD) cases, enhancing the reliability and depth of our comparative analysis.

The inclusion criteria were as follows: any indication for pancreaticoduodenectomy, a robotic surgical approach, age over 18 years and high difficulty.

High difficulty was defined according to PD-ROBOSCORE considering the following factors: high body mass index (BMI) (male, female), right hepatic artery (RHA) from superior mesenteric artery (SMA), ≤ 3 mm pancreatic duct, uncinate process tumor location, and previous major abdominal surgery. Difficulty score was estimated by using an online calculator (https://yuboski.github.io/pd-roboscore/) for each patient [4]. As previously described, a score of more than 9 was defined as a high score.

For the purpose of this study, preoperative pancreatitis with collections at computed tomography (CT) scan, preoperative cholangitis requiring antibiotic treatment or biliary stenting, and tumor contact with portal vein and/or superior mesenteric vein (PV-SMV) were also evaluated: although these factors are not included in the PD-ROBOSCORE, there are several publications that demonstrated their impact on intraoperative difficulty in pancreatic surgery [4, 6].

The primary outcome was the comparison of 90-day complications between RPD and OPD patients. Secondary outcomes included operating time, blood loss, and postoperative pancreatic fistula (POPF).

Surgical technique was performed as previously described by Giulianotti et al. with 17 phases [13]. First, gastrocolic ligament is opened, gastroepiploic vessels are divided, and right-colonic flexure is taken down to expose the duodenum. Then, a wide Kocher maneuver is performed allowing the visualization of left renal vein and superior mesenteric artery. After the hepatic hilum is explored, right‐gastric‐artery is divided, the gallbladder is taken down and common bile duct is divided. Stomach is divided pre-pyloric. Then gastroduodenal artery is divided. After, the first jejunal limb is selected and transected, the mesenteric detachment from the jejunum is carried out. From the right side of the aorta, the duodenum is passed to the right of the aorto-mesenteric axis. Then, the pancreas is transected with the Harmonic. Then the uncinate process is dissected while pulling superior mesenteric vein (SMV) slightly to the left, the dissection is done in a caudal-to-caphalic direction completing pancreaticoduodenectomy by carefully dissecting the mesopancreas. A Child limb is used for reconstruction. First, pancreaticojejunostomy is performed with a stent protecting the duct to mucosa anastomosis. Then hepatico-jejunostomy is performed with 4/0 absorbable barbade suture. Finally, side to side gastro-jejunal anastomosis is performed by endoscopic stapler.

In addition, routine biodegradable biliary stents were used in hepaticojejunostomy and gastroduodenal artery stump protection with Teres ligament was always performed, as previously published by our center in open surgery [3]. All patients underwent preoperative cardiopulmonary exercise test and prehabilitation according to risk assessment.

In our Center, pancreatic specimens are examined according to the protocols provided by the College of American Pathologists. The guidelines from the College of American Pathologists and final reports are issued following the cancer protocol templates [7, 8].

All the operations were performed by a single surgeon (FA) with large previous experience both in open (> 1000) and minimally invasive (> 300) hepato-pancreato-biliary operations.

Complications were defined according to Clavien-Dindo classification and Comprehensive Complication index (CCI) [9].

Pancreatic, biliary and chyle leaks were defined according to the International Study Group of Pancreatic and Liver Surgery criteria [10–12]. Pure bile leaks were also identified based on the co-occurrence of pancreatic fistula.

The postoperative period was managed as previously described. Following hospital discharge, all patients were followed up clinically at 1 month, and then every 6 months with abdominal imaging (CT or magnetic resonance imaging [MRI]) and blood tests including tumor markers.

All categorical data are presented as the number of cases and percentages. Chi-square and Fisher’s exact tests, when appropriate, were used to compare proportional data. Continuous nonparametric data were expressed as the median with interquartile range (IQR), while parametric data were expressed as the mean with standard deviation (SD). The Mann–Whitney U test was used for comparing nonparametric variables, and the t test was used for parametric continuous variables. All the tests were 2-sided, and the threshold of significance was set at p < 0.05. Statistical analyses were performed using Statistical Package for Social Sciences software (IBM SPSS Statistics, Version 27 for Macintosh; IBM Corp., Armonk, NY, USA). The results were reported according to the strengthening of the reporting of observational studies in epidemiology (STROBE) statement guidelines [13].

This research was conducted in accordance with the protocol, the principles of the latest revised version of the Declaration of Helsinki (Seoul, 2008), the standards of Good Clinical Practice as outlined by the Harmonized Tripartite Standards of the ICH for Good Clinical Practice (1996), and the guidelines for Good Epidemiological Practice (http://www.ieaweb.org/GEP07.htm). The study was evaluated and approved by the Clinical Research Ethics Committee (CEIC) of the Hospital Clinic of Barcelona (HCB/2024/0924). The study was evaluated and approved by the Clinical Research Ethics Committee (CEIC) of the Hospital Clinic of Barcelona (HCB/2024/0924).

## Results

**Fig. 1:**
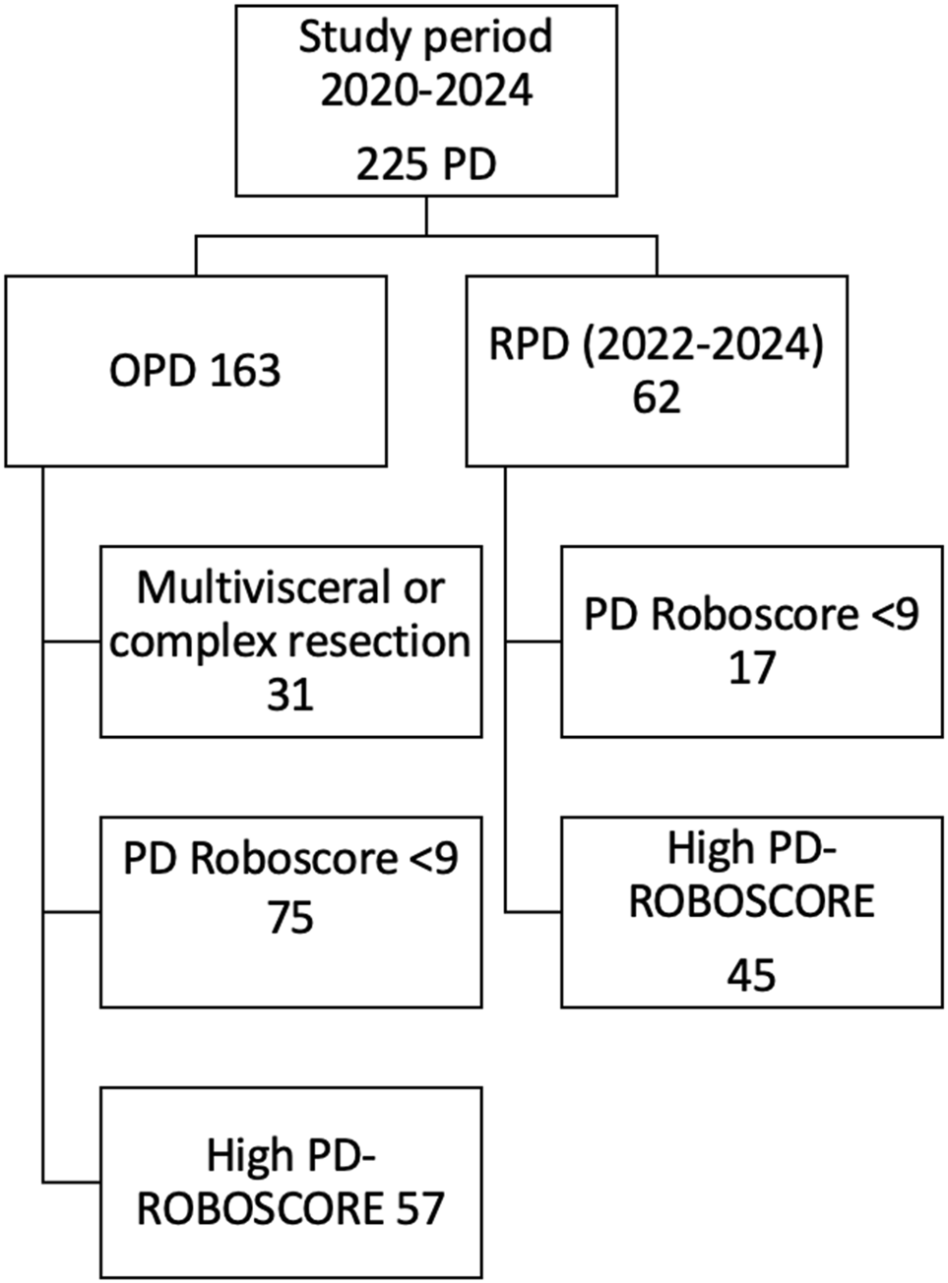
Flowchart of patient’s selection. *OPD* open pancreaticoduodenectomy, *RPD* robotic pancreaticoduodenectomy, *PD* pancreaticoduodenectomy

A total of 225 pancreaticoduodenectomies were performed during the study duration. The RDP robotic program at our Institution started in January 2022. Out of 62, 45 RPD had a high PD-ROBOSCORE and therefore were included in this study. These patients were compared with a retrospective cohort of 57 out of 163 OPD patients who had a high PD-ROBOSCORE. A patient’s flowchart is shown in Fig. 1. Patients’ characteristics are described in Table 1.

**Table 1:** Patients characteristics

	RPD (n = 45)	OPD (n = 57)	P
Age, y, median (IQR)	68 (64–72)	65 (61–68)	0.257
BMI, kg/m^2^, median (IQR)	27 (24–27)	28 (27–29)	0.132
Females, n (%)	18 (40.0)	17 (29.8)	0.153
Males with BMI ≥ 25 kg/m2	18 (66.6)	31 (77,5)	
Females with BMI ≥ 30 kg/m2	9 (50.0)	6 (35.2)	
High BMI, n (%)	27 (60.0)	37 (65.0)	0.726
ASA ≥ 3, n (%)	30 (66.6)	19 (33.3)	0.398
Type of tumor, n (%)			
PDAC	19 (42.2)	23 (40.3)	0.863
Distal cholangiocarcinoma	12 (26.7)	15 (26.3)	
Ampullary cancer	6 (13.3)	7 (12.3)	
Others	8 (17.7)	12 (21.0)	
Soft pancreas	34 (75.5)	27 (67.5)	0.401
Common or RHA from SMA	14 (31.1)	8 (14.0)	0.532
1–2 mm pancreatic duct, n (%)	45 (100)	52 (91.2)	0.782
Uncinate tumor location, n (%)	11 (24.4)	9 (15.7)	0.232
Previous abdominal surgery, n (%)	18 (40.0)	15 (26.3)	0.175
Neoadjuvant treatment, n (%)	2 (4.4)	3 (5.2)	0.633
Preoperative pancreatitis, n (%)	12 (26.7)	10 (17.5)	0.384
Preoperative cholangitis, n (%)	21 (46.7)	24 (42.1)	0.687
Tumor contact with PV-SMV, n (%)	5 (11.1)	5 (8.7)	0.779
Median difficulty score, n (%)	10 (9–12)	9 (9 – 10)	0.145
≥ 2 difficulty factors, n (%)	55%	42%	0.272

*RPD* robotic pancreaticoduodenectomy, *OPD* open pancreaticoduodenectomy, *BMI* body mass index, *ASA* American Society of Anesthesiologists (ASA) physical status classification, *PDAC* pancreatic adenocarcinoma, *RHA* right hepatic artery, *SMA* superior mesenteric artery, *PV* portal vein, *SMV*, superior mesenteric vein

Median age was 68.5 years (68 RPD vs 65 OPD; p = 0.25), median BMI was 27 kg/m^2^ (27 RPD vs 28 OPD; p = 0.13) and 65.6% of patients were male (60.0% RPD vs 70.2% OPD; p = 0.15).

When analyzing the full cohort, preoperative pancreatitis was not associated with blood loss higher than 400 ml (pancreatitis 13.3% vs no-pancreatitis 14.3%, p = 0.885), prolonged operative time (pancreatitis 398 min vs no-pancreatitis 391 min, p = 0.492); preoperative cholangitis was not associated with prolonged operative time (cholangitis 414 min vs no-cholangitis 363 min, p = 0.093) blood loss higher than 400 ml (cholangitis 18.9% vs no-cholangitis 6.8%, p = 0.158). High BMI showed not statistically significance with blood loss higher than 400 ml (0% vs 18.3%, p = 0.140) and operative time (399 min vs 391; p = 0.862). Intraoperative data are shown in Table 2.

**Table 2:** Intraoperative outcomes

	RPD (n = 45)	OPD (n = 57)	P
Operative time, min., median (IQR)	480 (400–575)	312 (258–367)	< 0.001
Blood loss > 400 ml, n (%)	0 (0)	13 (22.8)	0.02
Portomesenteric venous resection, n (%)	4 (8.8)	3 (5.2)	0.758
Conversion, n (%)	10 (22.2)	NA	NA
Tumor size, mm, median (IQR)	24 (17–33)	26 (18–32)	0.762
R0 resection in PDAC*, n (%)	15 (78.9)	18 (78.2)	0.825
Lymph node harvest, median, (IQR)	13 (10–19)	12 (10–17)	0.889
Lymph node harvest, PDAC*, median, (IQR)	17 (15–21)	16 (15–19)	0.865

IQR, Interquartile range; R0, microscopically margin-negative resection; *PDAC* pancreatic adenocarcinoma

There were no emergency conversions in our series. When analyzing conversion in RPD the only difficulty factor associated with conversion was tumor contact with PV-SMV (80.0% borderline resectable [BL] vs 13.0% no-BL; p = 0.04). Preoperative pancreatitis, preoperative cholangitis or high BMI showed no statistical significance.

Postoperative outcomes were analyzed: POPF occurred in 37.2% (40.0% RPD vs 35.1% OPD; p = 0.67), CD ≥ 3 was 25.4% (28.8% RPD vs 22.8% OPD; p = 0.47), median CCI was 20.9 (20.5 RPD vs 20.9 OPD; p = 0.75), reoperation rate was 17.6% (15.5% in RPD vs 19.3% in OPD; p = 0.48). Hospital stay was 15 days (16 RPD vs 13 OPD (p = 0.58). Amongst patients developing POPF, 29 (76.3%) had soft pancreas, 32 (84.2%) had a pancreatic duct diameter ≤ 2 mm and 36 (97.2%) had BMI ≥ 25.

Preoperative pancreatitis (pancreatitis 40.0% vs no-pancreatitis 20.4%, p = 0.119), preoperative cholangitis (cholangitis 27.6% vs no-cholangitis 24.3%, p = 0.764), and tumor contact with the PV-SMV (venous contact 25.0% vs no-contact 26.8%, p = 0.885), did not show any statistically significant association with major complications. These data are shown in Table 3.

**Table 3:** Comparison of postoperative outcomes in open vs robotic high-difficulty PD patients

	RPD (n = 45)	OPD (n = 57)	P
POPF	18 (40.0)	20 (35.1)	0.668
• Grade B, n (%)	13 (28.9)	13 (22.8)	
• Grade C, n (%)	5 (11.1)	7 (12.3)	
DGE (grade B/C), n (%)	9 (20.0)	10 (17.5)	0.336
PPH (grade B/C), n (%)	4 (8.8)	3 (7.5)	0.398
Bile leak (grade B/C), n (%)	0 (0)	0 (0)	–
Chyle leak (grade B/C), n (%)	0 (0)	0 (0)	–
CD ≥ 3	13 (28.8)	13 (22.8)	0.477
Median CCI	20.5	20.9	0.752
Reoperation	7 (15.5)	11 (19.3)	0.486
Hospital stay, days median, IQR	16 (7–26)	13 (10–21)	0.583
Readmission within 30 days n (%)	6 (13.3)	10 (17.5)	0.635
In-hospital mortality or 30 day mortality, n (%)	1 (2.2)	2 (3.5)	0.836

*POPF* postoperative pancreatic fistula, *DGE* delayed gastric emptying, *RPD*, robotic pancreatoduodenectomy, *OPD* open pancreaticoduodenectomy, *PDAC* pancreatic ductal adenocarcinoma, *PPH* postpancreatectomy hemorrhage, *CD* Clavien-Dindo classification, *CCI* comprehensive complication index, *IQR* interquartile range

## Discussion

Our study demonstrated that robotic pancreaticoduodenectomy (RPD) is not inferior to open pancreaticoduodenectomy (OPD), even in patients with high PD-ROBOSCORE. Despite a longer operative time and higher conversion rates, RPD outcomes were comparable to OPD. Robotic surgery, with its enhanced visualization and precision, offers advantages such as reduced blood loss and faster recovery while addressing technical challenges in delicate dissections near vital structures [14]. However, it requires significant surgical expertise, with an estimated 84 cases needed for proficiency.

PD-ROBOSCORE, designed to assess RPD difficulty, aids in preoperative planning and identifying high-risk cases [4]. Patients with high scores are at increased risk of complications like POPF and mortality. However, PD-ROBOSCORE has limitations, including its lack of consideration for surgeon experience and patient-specific comorbidities, making it less applicable in centers without advanced robotic programs [15]. Despite these drawbacks, the score remains a valuable tool for evaluating surgical complexity and outcomes [14].

In our study, there were no emergency conversions, but tumor contact with the PV or SMV significantly predicted conversion (80.0% with contact vs. 13.0% without; p = 0.04).

Postoperative outcomes, including POPF, Clavien-Dindo grade ≥ 3 complications, composite complication index (CCI), reoperation rate, and hospital stay, were similar between RPD and OPD. POPF occurred in 37.2% of patients (40.0% RPD vs. 35.1% OPD; p = 0.67), and major complications were observed in 25.4% (28.8% RPD vs. 22.8% OPD; p = 0.47). Hospital stay and reoperation rates also showed no significant differences, confirming that RPD does not increase the risk of complications, even in technically complex cases.

Specific patient-related factors were strongly associated with POPF. Among patients developing this complication, 76.3% had soft pancreases, 84.2% had pancreatic duct diameters ≤ 2 mm, and 97.2% had BMI ≥ 25. These well-known risk factors significantly contribute to complications, regardless of the surgical approach. High BMI increases intra-abdominal fat, complicating dissection and raising infection risks. Soft pancreatic texture and small ducts challenge secure anastomoses, heightening the risk of leaks. While RPD’s precision may help navigate these issues, it cannot entirely mitigate anatomical risks.

Preoperative pancreatitis and cholangitis, though not statistically associated with major complications in our study, pose technical challenges. Pancreatitis can lead to adhesions, fibrosis, and increased bleeding risk, while cholangitis-related tissue fragility and scarring complicate dissections and reconstructions [6]. Preoperative biliary stenting, often required for cholangitis, can also introduce additional risks. These findings highlight the importance of careful preoperative planning, particularly in high PD-ROBOSCORE patients.

Tumor contact with PV-SMV significantly increased conversion rates but did not worsen perioperative outcomes. In cases of vascular involvement, advanced skills are needed for safe dissection and potential vascular reconstruction. Our findings suggest that RPD remains a feasible option in such cases when performed by experienced surgeons.

Despite challenges, our results underscore that complications in high-risk patients often stem from patient-specific factors rather than procedural limitations. The similarity in outcomes between RPD and OPD, even in technically complex cases, supports the broader adoption of RPD in high-risk populations. Continued advancements in robotic technology and refinement of scoring systems like PD-ROBOSCORE will further enhance patient selection and surgical outcomes.

This study has several limitations. As a retrospective analysis, it relies on previously collected data, which may be incomplete, biased, or lack critical variables, potentially affecting the accuracy and reliability of the conclusions. Additionally, the small sample size increases the risk of statistical error, making it difficult to detect true effects or generalize findings to broader patient populations. Selection bias is another potential limitation, as the sample may not fully represent the wider patient demographic or clinical spectrum. Consequently, these findings should be interpreted with caution and validated through larger, prospective studies.

Moreover, many patients included in this study underwent surgery during the learning curve for robotic pancreaticoduodenectomy (RPD), which could have influenced the outcomes. However, since the results between the RPD and open pancreaticoduodenectomy (OPD) groups were comparable, it is unlikely that the benefits of the robotic approach were overestimated. While the 22% conversion rate in patients with high PD-ROBOSCORE may seem high, it is important to note that emergency conversions were entirely avoided in our series. Prioritizing planned rather than emergency conversions likely contributed to the higher overall conversion rate but minimized intraoperative risks and ensured safer outcomes for patients.

In conclusion, patients with high PD-ROBOSCORE show compared outcomes regardless of the surgical approach and therefore RPD is not inferior to OPD. Ultimately, the decision between robotic or open surgery should depend on the surgeon’s skill, patient-specific factors, and the complexity of the procedure, as both methods have situational advantages. Larger studies are needed to validate these findings.

## References

[1] Simon R (2021) Complications after pancreaticoduodenectomy. Surg Clin North Am 101(5):865–874. 10.1016/j.suc.2021.06.011 34537148 10.1016/j.suc.2021.06.011

[2] Liu R, Hilal MA, Besselink MG, Hackert T, Palanivelu C, Zhao Y, He J, Boggi U, Jang JY, Panaro F, Goh BK, Efanov M, Nagakawa Y, Kim HJ, Yin X, Zhao Z, Shyr YM, Iyer S, Kakiashvili E, Han HS, Lee JH, Croner R, Wang SE, Marino MV, Prasad A, Wang W, He S (2024) International consensus guidelines on robotic pancreatic surgery in 2023. Hepatobiliary Surg Nutr 13(1):89–104 38322212 10.21037/hbsn-23-132 PMC10839730

[3] Ausania F, Martínez-Pérez A, Senra Del Rio P, Borin A, Melendez R, Casal-Nuñez JE (2021) Multifactorial mitigation strategy to reduce clinically relevant pancreatic fistula in high-risk pancreatojejunostomy following pancreaticoduodenectomy. Pancreatology 21(2):466–472. 10.1016/j.pan.2020.12.019 33454209 10.1016/j.pan.2020.12.019

[4] Napoli N, Cacace C, Kauffmann EF, Jones L, Ginesini M, Gianfaldoni C, Salamone A, Asta F, Ripolli A, Di Dato A, Busch OR, Cappelle ML, Chao YJ, de Wilde RF, Hackert T, Jang JY, Koerkamp BG, Kwon W, Lips D, Luyer MDP (2023) The PD-ROBOSCORE: a difficulty score for robotic pancreatoduodenectomy. Surgery 173(6):1438–1446. 10.1016/j.surg.2023.02.020 36973127 10.1016/j.surg.2023.02.020

[5] Callery MP, Pratt WB, Kent TS, Chaikof EL, Vollmer CM Jr (2013) A prospectively validated clinical risk score accurately predicts pancreatic fistula after pancreatoduodenectomy. J Am Coll Surg 216(1):1–14. 10.1016/j.jamcollsurg.2012.09.002 23122535 10.1016/j.jamcollsurg.2012.09.002

[6] Chen YH, Xie SM, Zhang H, Tan CL, Ke NW, Mai G, Liu XB (2015) Clinical impact of preoperative acute pancreatitis in patients who undergo pancreaticoduodenectomy for periampullary tumors. World J Gastroenterol 21(22):6937–6943. 10.3748/wjg.v21.i22.6937 26078571 10.3748/wjg.v21.i22.6937 PMC4462735

[7] Burgart LJ, Chopp WV, Jain D (2021) Protocol for the examination of specimens from patients with carcinoma of the pancreas. College of American Pathologists (CAP) [Revised 5 June 2023]. https://www.cap.org/protocols-and-guidelines/cancer-reporting-tools/cancer-protocol-templates

[8] Amin MB, Greene FL, Edge SB, Compton CC, Gershenwald JE, Brookland RK, Meyer L, Gress DM, Byrd DR, Winchester DP (2017) The eighth edition AJCC cancer staging manual: continuing to build a bridge from a population-based to a more personalized approach to cancer staging. Cancer J Clin. 67(2):93–9. 10.3322/caac.21388 10.3322/caac.21388 28094848

[9] Clavien PA, Barkun J, de Oliveira ML, Vauthey JN, Dindo D, Schulick RD, de Santibañes E, Pekolj J, Slankamenac K, Bassi C, Graf R, Vonlanthen R, Padbury R, Cameron JL, Makuuchi M (2009) The Clavien-Dindo classification of surgical complications: five-year experience. Ann Surg 250(2):187–196. 10.1097/SLA.0b013e3181b13ca2 19638912 10.1097/SLA.0b013e3181b13ca2

[10] Bassi C, Marchegiani G, Dervenis C, Sarr M, Abu Hilal M, Adham M, Allen P, Andersson R, Asbun HJ, Besselink MG, Conlon K, Del Chiaro M, Falconi M, Fernandez-Cruz L, Fernandez-Del Castillo C, Fingerhut A, Friess H, Gouma DJ, Hackert T, Izbicki J (2017) International study group on pancreatic surgery (ISGPS) the 2016 update of the international study group (ISGPS) definition and grading of postoperative pancreatic fistula: 11 years after. Surgery 161(3):584–591. 10.1016/j.surg.2016.11.014 28040257 10.1016/j.surg.2016.11.014

[11] Mungroop TH, van Rijssen LB, van Klaveren D, Smits FJ, van Woerden V, Linnemann RJ, de Pastena M, Klompmaker S, Marchegiani G, Ecker BL, van Dieren S, Bonsing B, Busch OR, van Dam RM, Erdmann J, van Eijck CH, Gerhards MF, van Goor H, van der Harst E, de Hingh IH (2019) Alternative fistula risk score for pancreatoduodenectomy (a-FRS): design and international external validation. Ann Surg 269(5):937–943. 10.1097/SLA.0000000000002620 29240007 10.1097/SLA.0000000000002620

[12] Brooke-Smith M, Figueras J, Ullah S, Rees M, Vauthey JN, Hugh TJ, Garden OJ, Fan ST, Crawford M, Makuuchi M, Yokoyama Y, Büchler M, Weitz J, Padbury R (2015) Prospective evaluation of the international study group for liver surgery definition of bile leak after a liver resection and the role of routine operative drainage: an international multicentre study. HPB (Oxford) 17(1):46–51. 10.1111/hpb.12322 25059275 10.1111/hpb.12322 PMC4266440

[13] von Elm E, Altman DG, Egger M, Pocock SJ, Gøtzsche PC, Vandenbroucke JP (2007) The strengthening the reporting of observational studies in epidemiology (STROBE) statement: guidelines for reporting observational studies. Lancet 370(9596):1453–1457. 10.1016/S0140-6736(07)61602-X 18064739 10.1016/S0140-6736(07)61602-X

[14] Ausania F, Landi F, Martinie JB, Vrochides D, Walsh M, Hossain SM, White S, Prabakaran V, Melstrom LG, Fong Y, Butturini G, Bignotto L, Valle V, Bing Y, Xiu D, Di Franco G, Sanchez-Bueno F, De Angelis N, Laurent A, Giuliani G, Giulianotti PC (2023) Robotic versus laparoscopic distal pancreatectomy in obese patients. Surg Endosc 37(11):8384–8393. 10.1007/s00464-023-10361-x 37715084 10.1007/s00464-023-10361-x PMC10615948

[15] Zwart MJ, Van Den Broek B, De Graaf N, Suurmeijer JA, Augustinus S, Te Riele WW, Van Santvoort HC, Hagendoorn J, Rinkes IH, Van Dam JL, Takagi K, Tran KT, Schreinemakers J, van der Schelling G, Wijsman JH, de Wilde RF, Festen S, Daams F, Luyer MD, de Hingh IHJT (2023) The feasibility, proficiency, and mastery learning curves in 635 robotic pancreatoduodenectomies following a multicenter training program: “standing on the shoulders of giants.” Ann Surg 278(6):e1232–e1241. 10.1097/SLA.0000000000005928 37288547 10.1097/SLA.0000000000005928 PMC10631507

